# Pre‐chewing for weaning: A traditional method for allergy prevention? Rationale, study design, and methods of the open‐label trial—Solids‐by‐Kiss

**DOI:** 10.1111/pai.70147

**Published:** 2025-08-29

**Authors:** Birgit Ahrens, Lara Meixner, Meral Sturmfels, Birgit Kalb, Anna Fischl, Falk Schwendicke, Katharina Blumchen, Andreas Fickenscher, Laura Schäfer, Thomas Holzhauser, Sabine Schnadt, Kirsten Beyer

**Affiliations:** ^1^ Department of Paediatrics, Division of Pneumology, Allergology, Infectious Diseases and Gastroenterology Goethe University Frankfurt Frankfurt (Main) Germany; ^2^ Allergology Division Paul‐Ehrlich‐Institut Langen Germany; ^3^ Department of Paediatric Respiratory Medicine, Immunology and Critical Care Medicine Charité – Universitätsmedizin Berlin, corporate member of Freie Universität Berlin and Humboldt‐Universität zu Berlin Berlin Germany; ^4^ Conservative Dentistry and Periodontology LMU Klinikum, LMU Munich Munich Germany; ^5^ Fickenschers Backhaus GmbH Münchberg Germany; ^6^ Deutscher Allergie Und Asthmabund e.V Mönchengladbach Germany; ^7^ German Center for Child and Adolescent Health (DZKJ) Partner Site Charité Universität Berlin Berlin Germany

**Keywords:** food allergy, microbiome, premastication (pre‐chewing), prevention

## Abstract

**Background:**

The increase of allergies is attributed to a multi‐factorial pathogenesis. Multi‐gene traits, as well as different environmental factors, are regarded as responsible for shaping the development of atopic entities. The microbiome's composition in particular seems to play a decisive role in allergy prevention and immunological defense. Another key factor is attributed to the early infant's diet. This trial aims to investigate the influence of an early introduction of processed allergenic foods via premastication, a common practice in human societies throughout the course of human evolution in which the caregiver pre‐chews solids before feeding them to the infant.

**Methods:**

Two parallel study groups of infants at risk for atopic diseases (*N* = 100) are planned to be included. Healthy caregivers of eligible infants can decide to participate by either (A) introducing solids with additional provision of a sugar‐free ground biscuit suitable for pre‐chewing that contains increasing amounts of hen's egg, cow's milk, peanut, and hazelnut proteins or (B) by introducing solids following current feeding recommendations. The primary endpoint will be the assessment of the diversity of the infant's intestinal microbiome. Secondary endpoints will include an assessment of the acceptance of the feeding habit, the development of food allergies or food sensitization, and infections, including potential caries development, in association with the composition of the infant's oral microbiome.

**Conclusion:**

In reflection of the multifactorial pathogenesis of atopic diseases, this trial combines key factors strongly assumed to act “allergy‐protective”: (1) microbial diversity supporting inflammatory resilience and (2) targeted dietary introduction of allergenic foods supporting food tolerance.

**Trial Registration:**

German Clinical Trials Register DRKS00027255. Registered on 29 April 2022.

**Abbreviations**
AE: Adverse Events
AESI: Adverse Events of Interest
ANCOVA: Analysis of Covariance
DBPCFC: Double‐Blind Placebo‐Controlled oral Food Challenge
DMFT: Decayed, missing, and filled teeth
EASI‐Score: Eczema Area and Severity Index Score
EAT Study: Enquiring About Tolerance Study
eCRF: electronic Case Report Form
FAS: Full Analysis Set
GCP: Good Clinical Practice
HEAP Study: Hen's Egg Allergy Prevention Study
ICDAS criteria: International Caries Detection and Assessment System
IgE: Immunoglobulin E
IgG4: Immunoglobulin G4
ITT: Intention‐To‐Treat
LEAP Study: Learning Early About Peanut Allergy Study
PETIT Study: Prevention of Egg Allergy with Tiny Amount Intake Study
POEM: Patient‐oriented Eczema Measure
PP: Per Protocol
PPS: Per Protocol Population Set
REDCap: Research Electronic Data Capture
SAE: Serious Adverse Event
SCORAD: Severity Scoring of Atopic Dermatitis
SPT: Skin Prick Test
STAR Study: Solids Timing for Allergy Reduction Study
TEWL: Transepidermal water loss

**Key message**

Premastication is a common feeding practice in human societies throughout the course of human evolution in which the mother pre‐chews especially solid foods before giving them to the infant. In this trial, we investigate the infant's microbiome as influenced by different feeding habits. An introduction of weaning food according to current recommendations is compared to an early introduction of processed allergenic foods via premastication.

## 1 INTRODUCTION

### 1.1 Background and rationale

There have been reports of an increase in food allergies for decades.^1^, ^2^ Currently, up to 10% of children worldwide suffer from IgE‐mediated food allergies^3^ with the most common being allergies to hen's eggs and cow's milk.^4^, ^5^ Additionally, of note are allergies to peanuts and tree nuts due to their association with severe allergic reactions and reduced spontaneous tolerance development.^3^

Allergies are attributed to a multi‐factorial pathogenesis. Multi‐gene traits and diverse environmental factors are regarded as key factors in shaping the development and/or severity of atopic entities.^6^, ^7^, ^8^, ^9^, ^10^, ^11^, ^12^, ^13^, ^14^

#### 1.1.1 Microbial diversity supports inflammatory resilience

In continuation of the hygiene hypothesis by Strachan et al.,^6^ various “pro‐allergy” factors have been described over the years, including: reduced household sizes, reduced infections,^7^ higher standards of personal hygiene, Caesarean section delivery,^8^ and limited exposure to pets.^9^ In contrast, a reduced risk of allergic disease development has been attributed to acquisition of hepatitis A/orofaecal microbes via personal contact^10^ or vaginal delivery.^11^ The individual and unique “special environmental experience” has been shown to be reflected in the gut microbiome, especially early in life.^12^ The host acquires mechanisms to distinguish between “microbial friends and foes” on the gut and other mucosal layers.^13^, ^14^, ^15^ A high level of bacterial diversity is suggested to promote the establishment of a tolerogenic immune response towards an inflammatory resilience.^13^

Building on these past findings, we want to analyze if and how the infant's microbiome will be shaped by a traditional infant feeding practice known as premastication.^16^ In this practice, the caregiver pre‐chews food for the infant before feeding it to them and therefore transmits the parental/caregiver oral microbiome.

#### 1.1.2 Early dietary intervention in the development of food allergies

The manifestation of food allergies typically begins in early infancy.^3^, ^4^, ^5^, ^17^, ^18^, ^19^, ^20^, ^21^, ^22^, ^23^ The particular focus at this time in life is on the “highly allergenic” foods (like cow's milk, hen's egg, peanut, tree nuts): when they will be introduced (“window of opportunity”), how much they are consumed and how often, or the way they are processed (e.g., raw, heated, hydrolysed). Key studies on this topic include the LEAP trial^22^ (UK), which focused on early peanut introduction, and the EAT study^23^ (UK), which examined the allergy preventive effect of introducing six allergenic foods to breastfed infants starting at 3 months of age. Furthermore, the HEAP (Germany),^18^ STAR (Australia),^19^ and PETIT (Japan)^20^ trials analyzed the introduction of different (and increasing) amounts of hen's egg protein, utilizing either cooked/heated or raw/pasteurized egg protein. A summary of the findings potentially leads to the conclusion that early dietary introduction and the frequent consumption of allergenic foods are regarded as beneficial.^17^, ^21^ Hen's eggs should be introduced thoroughly heated, but not raw.^17^, ^21^

We now want to analyze the parallel introduction of increasing amounts of four “highly allergenic” foods, all processed (baked) in the form of a sugar‐free ground biscuit that is offered to the mother/caregiver for pre‐chewing.

Our aim in using this method is to combine both factors outlined above as they are assumed to act in an “allergy‐protective” manner by: (1) strengthening the microbial diversity that supports inflammatory resilience by transmitting the mother's oral microbiome and (2) targeting dietary introduction of processed allergenic foods that support food tolerance.

We choose an open study design in order to recruit caregivers with a high level of assumed compliance if they choose to take part in the pre‐chewing group.

This is an abridged protocol based on protocol version 2.0 dated 04 October 2021. The approach is prepared in consideration of the Standard Protocol Items: Recommendations for Interventional Trials (SPIRIT) recommendations for interventional trials.^24^

### 1.2 Objectives

The primary objective is to compare the impact of different feeding habits on the diversity of the infant's intestinal microbiome. Infants will be introduced to solid food, either with reference to current recommendations as a reference group or by utilizing the traditional feeding practice, by pre‐chewing a sugar‐free ground biscuit in parallel to other weaning foods and breastfeeding (non‐randomized). Secondary objectives focus on the assessment of acceptance of the feeding habit; the development of allergic reactions, including food allergies and food sensitization; and infections, including observation of potential caries development, gastrointestinal problems, and thriving aspects.

### 1.3 Trial design

**FIGURE 1:**
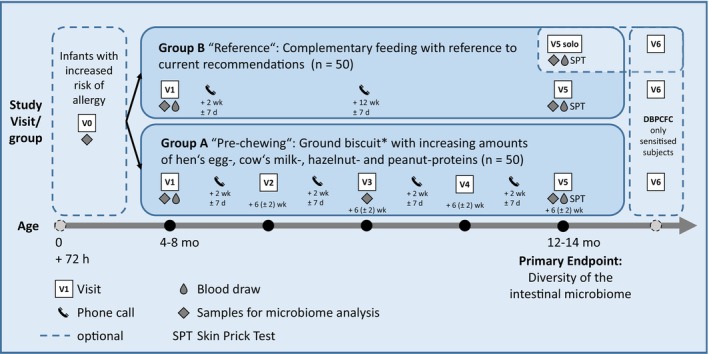
The sugar‐free ground biscuit* contains defined amounts of hen's egg, cow's milk, peanut, and hazelnut proteins, which will be increased from levels around ED05 in four steps by a factor of five at each step. In Group A, the first (pre‐chewed) portion will be given under medical supervision at the study site (V1, 4–8 months of age) followed by feeding at home once daily. At V2: 6 weeks after V1 ± 14 days, infants will receive the new ground biscuit, with protein from each of the four food allergens increased by a factor of five; the same will happen at V3 (6 weeks after V2 ± 14 days) and V4 (6 weeks after V3 ± 14 days). The first portion of each ground biscuit with increased protein content will be given at the study site under direct, two‐hour medical observation. * The ground biscuit will be analyzed on microbiological criteria for foodstuffs in accordance with COMMISSION REGULATION (EC) No. 2073/2005. d, days; DBPCFC, double blind placebo controlled food challenge; h, hours; wk, weeks.

Solids‐by‐Kiss is designed as an exploratory, open, two‐centre trial with two parallel study groups (Group A and B) in infants with risk for atopic diseases (Figure 1).

The trial is part of the collaborative project Solids‐by‐Kiss (Please see “Funding”). Subprojects address (1) development and production of the “test‐biscuit”; (2) its qualitative and quantitative analytical verification; (3) consumer communication including allergen‐labelling; and (4) diagnostic methods for microbiome analysis. Subprojects will be described separately.

## 2 METHODS: PARTICIPANTS, INTERVENTIONS, AND OUTCOMES

### 2.1 Study setting

All visits throughout the study will take place either in the study centres (V1–V5) or on the ward (V0: on the ward of the Division Obstetrics and Prenatal Medicine, Goethe University Frankfurt, Frankfurt am Main, Germany, and V6: on the ward of the Department of Pediatrics, Division of Pneumology, Allergology, Infectious Diseases and Gastroenterology, Goethe University Frankfurt, or of the Department of Pediatric Respiratory Medicine, Immunology and Critical Care Medicine, Charité – Universitätsmedizin Berlin).

### 2.2 Eligibility criteria

Only healthy participants can take part in the study. The detailed inclusion and exclusion criteria are listed in Table 1.

**TABLE 1:** Inclusion and exclusion criteria for Group A or Group B.

Inclusion criteria	Exclusion criteria
For the infant	For the infant
Healthy, full‐term born infants Positive family history for atopic diseases (at least one parent or sibling suffers from atopic diseases such as atopic eczema, food allergy, allergic rhino conjunctivitis, allergic asthma)	Previously consumed hen's egg, peanut, or hazelnut^a^ Suffering from a medically diagnosed wheat allergy
For the caregiver	For the caregiver
No food allergies to hen's egg, cow's milk, peanut, wheat, or hazelnut No (acute, sub‐chronic, or chronic) infectious diseases (including caries) according to physician's decision Mental and linguistic capability to follow the protocol requirements	Any reported disease or medical condition (for example, but not limited to, severe concurrent disease, infection, open wounds in the mouth, comorbidity, for example, acute trauma, serious oncological disease, serious renal disease, relevant cardiovascular disease, autoimmune diseases) or reasons that may, in the opinion of the study physician, interfere with the ability to participate in the study, cause undue risk, or complicate the interpretation of data concerning child or caregiver

^a^ Previous consumption of cow's milk, for example, as infant formula, is allowed since infant formula is commonly produced using cow's milk and formula‐fed infants should not be excluded.

Please see Appensix S1 for further information.

### 2.3 Intervention

#### 2.3.1 Intervention description

Mothers of both groups will be encouraged to continue breastfeeding while weaning foods are introduced stepwise to the infant's diet. Parents will decide in advance which group they would like to join, and—depending on the child's individual development—when first complementary foods are to be introduced (Figure 1). All participants will be informed equally regarding current feeding recommendations.^17^, ^21^

All Group A participants will visit the study site at five timepoints (V1–V5). Group B participants will visit the study site at two timepoints (V1 and V5). Blood samples will be collected at V1 and V5; SPT will be performed at V5 only. Samples for microbiome analysis will be taken at indicated visits. Telephone calls are scheduled 2 weeks after V1, V2, V3, and V4 (Group A) and 2 weeks after V1 as well as one interim phone call at week 12 (Group B).

All caregivers will be encouraged to keep a weekly diary recording frequency of pre‐chewing, tolerability, and palatability of solids introduction, as well as any adverse events, concomitant medication, and diet.

V0 (collection of stools + skin swabs) and V6 (enhanced diagnostic in sensitized subjects, including oral food challenges) are optional.

V5 solo consists only of V5 (and optional V6); participants will not have been enrolled before.

Group A – “Interventional/Pre‐chewing Group”: The mother (or one other previously determined caregiver) of an eligible infant will receive a sugar‐free ground biscuit once the infant starts eating complementary foods. The biscuit is ground to ensure homogeneity of the amount of included food proteins. These amounts will be increased by a factor of five in four stages, each lasting 6 weeks ±14 days, V1–V4 (Table 2). The actual amount of total protein of the allergenic food in the first dose at V1 (Table 2) starts slightly below calculated discrete ED05 values for the correspondent food allergens,^25^ which are 2.1 mg peanut protein, 3.5 mg hazelnut protein, 2.3 mg hen's egg protein, and 2.4 mg cow's milk protein. Further ingredients of the biscuit are wheat flour type 550 and margarine processed with rapeseed oil.

**TABLE 2:** Content of Peanut, Hazelnut, Hen's Egg, and Cow's Milk protein in the ground biscuit.

Group A	Visit 1–Visit 2	Visit 2–Visit 3	Visit 3–Visit 4	Visit 4–Visit 5/6
Amount of total protein (mg/3 g) (Per actual weight of the ingredients (e.g., peanut flour) and determination of the protein content (according to Kjeldahl) of the ingredients (e.g., peanut flour with 49.64% protein))				
Peanut	1.8	9.1	42.3	157
Hazelnut	2.8	13.5	63	234
Hen's egg	1.9	9.3	43.2	161
Cow's milk	2.1	10.1	47.2	176

*Note*: The actual amount of allergen in a 3 g portion, that is, the amount of total protein (e.g., peanut total protein) of the allergenic food (e.g., peanut), was calculated on the basis of actual weight of all ingredients while also considering the experimentally determined total protein content (according to total nitrogen Kjeldahl method) of the individual ingredients (e.g., partially defatted peanut flour with 49.64% total protein) and the loss of water by baking. Details of the calculation of allergen doses and comparability of allergen content and assessment of allergenicity of subsequent batches of the ground biscuit during the course of the project will be published separately after all batches have been produced and analyzed in vitro. This information will include relative quantitative data obtained by allergen‐specific ELISA^26^ for verification of relative increments of doses V1–V4 as well as functional mediator release from passively sensitized rat basophils^27^, ^28^ for assessment of relative allergenicity of the doses V1–V4.

Each dose is represented by a 3 g portion of the ground biscuit (measured using a measuring spoon) that should be given to the child once daily at least four times a week. The ground product is suggested to be pre‐chewed for 15–20 s by the determined caregiver until a smooth and soft puree portion is gained, which can be passed mouth to mouth or using a spoon, depending on individual preference. The first portion of each ground biscuit (with increased protein content) will be given at the study site under direct, two‐hour medical observation. It is important that the instructions for administering the pre‐chewed ground biscuit are followed, for example, the caregiver must not pre‐chew if they have an infection or an open wound in the mouth or if they have brushed their teeth or smoked beforehand. After receiving the last ground biscuit at V4, study participants will be invited for the end of study visit, V5.

Group B – Non‐randomized reference: Solids will be introduced with reference to current feeding recommendations^17^, ^21^ based on the parents' decision but without pre‐chewing. Study participants will be invited for the end of study visit (V5) around the child's first birthday.

V5 solo consists only of V5 in Group B (and optional V6); participants will not have been enrolled before. This group serves as an additional reference group, as participants will not have been informed (biased) about premastication before.

At V1 and V5, blood samples from the infant will be collected and stored for allergy diagnostics (including food‐specific IgE and IgG4 to hen's egg, cow's milk, peanut, hazelnut, Ara h 2, Cor a 14, and wheat).

At V5 (or V5 solo), infants will be skin prick tested with hen's egg, cow's milk, hazelnut, and peanut.

In case of the detection of a food‐specific sensitization (elevated food‐specific IgE (>0.1 kU/L) or positive Skin Prick Test, SPT (wheel size >3 mm)) at V5, a double‐blind, placebo‐controlled oral food challenge (DBPCFC) will be offered for evaluation of clinical relevance (optional visit V6). Consequently, parents will be advised to continue feeding the study product until the DBPCFC is performed.

Importantly, apart from the intervention with the ground biscuit in group A, there are no further dietary restrictions imposed by the study protocol for both of the study groups.

All participants may continue their usual medications, as well as those taken for any concomitant diseases, including wheezing and eczema, throughout the study. All subjects who will undergo a DBPCFC will be advised to discontinue oral antihistamines 3–5 days before their appointment. For a detailed description of all study procedures, see Table 3.

**TABLE 3:** Schedule of Events: (a) Group A and (b) Group B.

Visit/Phone call	Enrolment/Screening		Intervention period							V5	(V6)^a^	Unscheduled visit^b^
	V0 (optional)	V1	PC 1	V2	PC2	V3	PC3	V4	PC4			
(a) *Schedule of Events – Group A*												
Timepoint (week)		0	2 wks after V1	6 wks (±14 d)	2 wks after V2	12 wks (±14 d)	2 wks after V3	18 wks (±14 d)	2 wks after V4	24 wks (±14d)		
Enrolment												
Informed consent	x	X^e^										
Check eligibility	x	X^e^										
Assessments												
Demographics/subject characteristics	x	x								x		
Family characteristics	x	x		x		x		x		x		
Medication, nutrition, exposure to smoking during pregnancy	x	x										
Questionnaires Allergy (family members)	x	x										
Medical history, vaccination record	x	x		x		x		x		x		
Review AEs			x	x	x	x	x	x	x	x	x	x
Review concomitant medication	x	x	x	x	x	x	x	x	x	x	x	x
Nutritional characteristics^d^	x	x		x		x		x		x		x
Physical examination	x	x		x		x		x		x	x	x
SCORAD, EASI score		x		x		x		x		x	x	x
Palm lines documentation		x										
TEWL (Berlin)		x		x		x		x		x		
SPT										x		x
Stool scales^d^	x	x		x		x		x		x		
Postnatal hygiene score		x		x		x		x		x		
Pacifier questionnaire		x		x		x		x		x		
Palatability score^d^		x		x		x		x		x		
POEM score (Eczema)		x										
Skin swab infant (cheek)	x	x				x				x		
Skin swab caregiver (cheek)	x	x								x		
Skin swab caregiver (mamilla)	x	x				x				x		
Skin swab infant (body, interscapular)	x	x				x				x		
Oral swab infant		x				x				x		
Oral swab caregiver		x								x		
Stool sample infant	x	x				x				x		
Stool sample caregiver	x	x								x		
Blood draw		x								x	(x)	x
Dental status		X (Caregiver)								X (Child)		
Intervention												
Feeding of SP at site		x		x		x		x				x
Dispensation of SP		x		x		x		x		(x)^a^		x
Review compliance + tolerance of SP			x		x		x		x			x
OFC											x^a^	

Visit/Phone call	Enrolment/Screening		Reference period				V5	V5 solo^c^	(V6)^a^	Unscheduled Visit^b^
	V0 (optional)	V1	PC1		PC2					
(b) *Schedule of Events – Group B*										
Timepoint (week)		0	2 wks after V1		12 wks		24 wks (±14 d)			
Enrolment										
Informed consent	x	x^e^						x		
Check eligibility	x	x^e^						x		
Assessments										
Demographics/subject characteristics	x	x					x	x		x
Family characteristics	x	x					x	x		x
Medication, nutrition, exposure to smoking during pregnancy	x	x						x		
Questionnaires Allergy (family members)	x	x						x		
Medical history, vaccination record	x	x					x	x		x
Review AEs			x		x		x		x	x
Review concomitant medication	x	x	x		x		x	x	x	x
Nutritional characteristics^d^	x	x					x	x		x
Physical examination	x	x					x	x	x	x
SCORAD, EASI score		x					x	x	x	x
Palm lines documentation		x						x		
TEWL (Berlin)		x					x	x		x
SPT							x	x		x
Stool scale^d^	x	x					x	x		
Postnatal hygiene score		x					x	x		
Pacifier questionnaire		x					x	x		
Palatability score^d^		x						x		
POEM score (Eczema)		x						x		
Skin swab infant (cheek)	x	x					x	x		
Skin swab caregiver (cheek)	x	x					x	x		
Skin swab caregiver (mamilla)	x	x					x	x		
Skin swab infant (body, interscapular)	x	x					x	x		
Oral swab infant		x					x	x		
Oral swab caregiver		x					x	x		
Stool sample infant	x	x					x	x		
Stool sample caregiver	x	x					x	x		
Blood draw		x					x	x	(x)	x
Dental status		x (Caregiver)					x (Child)			
OFC									x	

*Note*: V0 and V6 (enhanced diagnostic in sensitized subjects) are optional.

Abbreviations: AE, adverse event; d, days; EASI, eczema area and severity index; OFC, oral food challenge; PC, phone call; POEM, patient‐oriented eczema measure; SP, Study/Interventional Product; SPT, skin prick testing; TEWL, transepidermal water loss measurement; V, visit; wks, weeks.

^a^ In case of sensitization until clinical relevance of the sensitization has been checked by an oral food challenge within 2 months after V5.

^b^ Any or all procedures performed at Unscheduled Visits.

^c^ V5 solo consists only of V5 in Group B (and optional V6); participants will not have been enrolled prior.

^d^ Monitored at V0/V1 and additionally for the duration of the study by collection of weekly diary entry.

^e^ If not already done at V0.

#### 2.3.2 Strategies to improve adherence to interventions and criteria for discontinuing or modifying allocated interventions

Please refer to Appendix S1.

### 2.4 Outcomes

#### 2.4.1 Primary endpoint

This study will compare the diversity of the intestinal microbiome in infants with risk for atopic diseases in Group A versus Group B after the introduction of solids (time point V5). The diversity will be measured via alpha diversity calculations; the Shannon diversity, the Gini‐Simpson index, or the evenness will be calculated.^29^

#### 2.4.2 Main secondary endpoints (for further endpoints, please see Appendix S1)

- Compliance assessment/adherence of parents/caregivers conducting pre‐chewing via questionnaire on performance and frequency: query on a weekly basis, if performed daily; 4–7 times per week; <3 times/week (compliance defined as at least 4 days/week ≙ 57%)
- Composition of the oral microbiome and frequency of infant caries by 1 year of age (V5) according to the International Caries Detection and Assessment System (ICDAS, a clinical scoring system allowing detection and assessment of caries lesions and caries experience^30^)
- Frequency of IgE‐mediated food allergies to at least one of the four foods (hen's egg, cow's milk, peanut, hazelnut) after intervention by 1 year of age (V5) between Groups A and B
- Allergen‐specific IgE and IgG4 to hen's egg, cow's milk, peanut, hazelnut, Ara h 2, Cor a 14, and wheat at V1 and V5 in comparison between groups.
- Cumulative occurrence (frequency and severity) of respiratory and gastrointestinal infections (including oral infections) in comparison between groups

### 2.5 Participant timeline

Please see Figure 1 and Table 3. Please refer also to Appensix S1 for correspondent information.

### 2.6 Recruitment

Please refer to Appendix S1 for recruitment information.

## 3 METHODS: DATA COLLECTION, MANAGEMENT, AND ANALYSIS

### 3.1 Data collection methods

#### 3.1.1 Clinical data

Written informed consent must be obtained at the screening visit, and inclusion and exclusion criteria will be reviewed. Information on demographics, subjects/family characteristics, relevant medical history, concomitant medication, and nutrition will be recorded. Questions on feeding habits, environmental factors, and family history will be repeated via a comprehensive questionnaire at different timepoints (V0, V1, and V5). Physical examination (including weight, and length measurements as well as SCORAD and EASI score when applicable^31^, ^32^, ^33^, ^34^) of the child will be carried out at every study visit besides V0, and information on individual use of pacifiers, thumb sucking, or other microbiome‐influencing aspects of personal hygiene will be collected using a routinely applied questionnaire at every study visit.

#### 3.1.2 Stool and saliva samples for microbiome analysis

Stool samples and skin swabs will be collected from the infants and the mothers at V0, V1, V3, and V5. The stool samples will be taken at home by the parents before the site visits. Collection of stool samples will be done using DNA/RNA Shield Fecal Collection Tubes (Zymo Research, Irvine, CA, USA). Our approach will be based on extraction and sequencing of V3–V4 regions of the 16SrRNA. The sequencing will be performed on a MiSeq platform (Illumina, San Diego, CA, USA) using V2 reagents (Illumina, San Diego, CA, USA). The sequencing length will be 2 × 250 bp. The diversity of the intestinal microbiome will be measured via alpha diversity calculations, which enable the comparison of the two groups with differing diets. Moreover, alpha diversity is a basis for other diversity calculations like the Shannon diversity, the Gini‐Simpson index, or the evenness, which will also be calculated.

Saliva samples (swab of the oral mucosa) will be collected from the infant and caregivers at V1 and at V5. Saliva samples will be obtained using Zymo Swabs (DNA/RNA Shield; Zymo Research, Irvine, CA, USA).

#### 3.1.3 Dental examination using telediagnostics

All (pre‐chewing) caregivers will receive a dental examination at V1 using telediagnostics. Caries lesions as well as missing and filled teeth (DMFT Index) will be assessed using the WHO protocol. Caries lesions will be assessed visually according to ICDAS without assessment of ICDAS code 1 (as drying the teeth will not be feasible using tele‐diagnostics).^30^ In case of existing caries lesions, the parent will be asked to treat corresponding lesions before starting the practice of pre‐chewing and will be invited for remaining V1 procedures and initiation of pre‐chewing after dental treatment only. The child's dental chart will be examined at V5 (end of study). Telediagnostics will be provided by an experienced dentist specifically trained upfront.

Please note the Appendix S1 for Continuation of *Clinical Assessment Methods and Clinical Outcome Definitions* on

- Skin swabs for microbiome analysis
- Allergy diagnostic including Skin Prick Test (SPT) and blood sampling
- Double‐Blind Placebo‐Controlled Food Challenge (DBPCFC)^35^
- Skin Barrier Permeability^36^

### 3.2 Data management

Please refer to Appendix S1 for information on data management.

### 3.3 Statistical methods

Diversity of the microbiome (primary endpoint) will be assessed utilizing alpha diversity calculations.^29^ The two study groups are compared using this diversity parameter. Moreover, based on the alpha diversity, other diversity indices like the Shannon diversity, the Gini‐Simpson index, or the evenness will be evaluated. No sample size calculation was performed due to the exploratory study design. *N* = 50 subjects per group is regarded as appropriate in order of magnitude of the few other studies in the context of pre‐chewing/saliva contact and/or microbiome analysis.^37^, ^38^, ^39^

In our explorative study, the primary efficacy analysis will be based on the full analysis set (FAS) and a secondary analysis will also be performed on the per protocol population (PPS) to assess the sensitivity of the analysis to the choice of analysis set. All safety analyses will be based on the full analysis set (FAS). The analysis will be performed without imputation of missing values. The significance level will be set to 0.05 (two‐sided). All other analyses will be considered explorative.

Univariable and multivariable logistic regression models will be performed for secondary binary endpoints (e.g., one diagnosed food allergy, food specific sensitisation, indices on microbiome diversity calculations). The analysis of secondary continuous endpoints (e.g., SCORAD, compliance over time) is conducted using analysis of covariance (ANCOVA). Child's sex, birth mode, number of siblings, pets, and other factors will be considered as covariates. Analysis will be carried out with the FAS. Based on the intention‐to‐treat (ITT) principle, the patients are analyzed according to the study group in which they started in.

Several explorative subcohort analyses (e.g., on antibiotic intake, infections) will be performed for the primary endpoint to find possible associations between covariates and diversity of the intestinal microbiome.

Selected secondary endpoints will also be analyzed with the PPS. The PPS is a subset of the FAS defined without participants with repeated insufficient consumption of the study product, defined as consumption <4 times/week (57%). As a general strategy, missing data will not be imputed in this study.

## 4 METHODS: MONITORING

Please refer to Appendix S1 for information on data “Monitoring”.

### 4.1 Harms

Any adverse event (AE) will be documented on paper (diary). Adverse events of special interest (AESIs) as well as serious adverse events (SAEs) will also be documented in the Research Electronic Data Capture, REDCap (Vanderbilt University in Nashville, USA). In case of an AESI, caregivers will be strictly advised to contact the study site as soon as possible. SAEs have to be reported within 48 h after knowledge of the event to the principal investigator. An SAE will be defined as any AE that fulfills at least one of the following criteria: results in death; is life‐threatening; requires inpatient hospitalization (longer than 24 h) or prolongation of existing hospitalization; or results in persistent or significant disability or incapacity.

The following are considered as AESIs:

- Objective immediate‐type allergic reactions within 2 hours of food exposure (either to the study product or due to exposure to other food allergens). Caregivers will be asked to call for medical help (emergency call), to document the symptoms in the subject's diary, and to call the study site before giving the next dose of the study product (Group A) in order to assess further details.
- Recurrent gastrointestinal symptoms/abdominal pain. Caregivers will be asked to document the symptoms in the subject's diary and to call the investigational site.
- Any form of new onset eczema should be reported immediately. An unscheduled site visit for visual examination and diagnosis of atopic dermatitis using modified Hanifin and Rajka criteria will be carried out according to investigators' discretion.^31^, ^32^, ^33^, ^34^ In case of a pre‐existing (at screening) or newly diagnosed atopic dermatitis, caregivers will be handed out an extra diary.

### 4.2 Auditing

Please refer to Appendix S1 for information on ‘auditing’.

## 5 DISCUSSION

In Solids‐by‐Kiss, we have designed a sugar‐free ground biscuit that contains increasing amounts of foods considered to be “highly allergenic”: hen's egg, cow's milk, peanut, and hazelnut. The biscuit is offered for pre‐chewing.

We are not aware of any prospective trials, especially not in the allergy area, analyzing the effect of pre‐chewing on the infant's microbial profile.^16^ Research on benefits or risks of premastication appears to be scarce and is mainly based on observations and case reports.^16^ Nevertheless, next to potential beneficial aspects, the transmission of pathogenic germs requires very careful consideration. As an example, for a long time the oral transfer of caries—regarded as an infectious disease induced by special bacteria like *Streptococcus mutans*—was recommended to be strictly avoided.^40^ In contrast, recent hypotheses support the development of a diverse, balanced oral microbiome for caries prevention.^41^, ^42^, ^43^, ^44^

It is important that the transmission of any diseases is reported, particularly when the person who pre‐chews the food is ill themselves. Just recently a case of HIV transmission by an HIV‐infected caregiver has been published.^45^

For this first exploratory approach, only healthy, full‐term born infants and healthy caregivers will be eligible for inclusion. Safety concerns are addressed as thoroughly as possible through constant referral to the precautionary measures in the instructions for administering the study product and close monitoring of infections or of any adverse reactions in the caregiver or the child. We track caries by examining the dental status of the caregiver and later of the infant and analyzing their oral microbiome pre and post intervention.

Our biggest concern is that we will not recruit enough caregivers who choose to take part in the pre‐chewing group. Premastication is not well known and is viewed rather critically in Germany.^46^ However, as our primary objective is to analyze whether the transmission of pre‐chewed foods for solid introduction has any influence on the infant's microbiome, we decided to choose an open‐study design despite the various limitations and the bias by the open selection of study groups. As data on the effect of pre‐chewing on microbiome analysis is rare, the estimated power is based on current publications that have in some way addressed the transfer of saliva,^38^ analyzed the oral microbiota in relation to allergy development,^39^ or analyzed its composition after premastication.^37^ Although the open design and the question of power are the biggest limitations, these factors also strengthen the outstanding position of this trial. This study will allow us to not only analyze the changes in microbiome composition (oral, skin, intestinal) over time, but also the influence of premastication for the first time. This exploratory first approach is built up carefully in order to identify danger signals early on. But we can also perhaps show that the exchange of parental saliva on the child is not “harmful” per se, but can also do “good”.

As final remark, it should be emphasized that this open trial will not be suitable for drawing recommendations on the prevention of cow's milk, hen's egg, peanut, and/or hazelnut allergies based on the outlined approach. This is particularly the case since no placebo biscuit (ground biscuit without the four food allergens) with or without pre‐chewing is included. Rather, the practicability and acceptance of the old feeding practice of premastication (if done at all, when and how often it is carried out) and its impact on the infant's microbiome will be assessed. Nevertheless, this study is a first step in pursuing the hypotheses on this combined approach and a potential utilization of premastication as an “allergy‐protective” puzzle piece for food allergy prevention.

## AUTHOR CONTRIBUTIONS

Birgit Ahrens and Kirsten Beyer were involved in the conception and trial design. Lara Meixner, Anna Fischl, and Katharina Blumchen contributed to the trial design. Involved in the study conduct and responsible for enrolling participants are Kirsten Beyer, Birgit Kalb, and Lara Meixner for Berlin, Anna Fischl, Meral Sturmfels, and Birgit Ahrens for Frankfurt. The manuscript was written by Birgit Ahrens with contributions by Anna Fischl, Birgit Kalb, and Kirsten Beyer; further contributions: Lara Meixner, Meral Sturmfels, Thomas Holzhauser, Laura Schäfer, Katharina Blumchen, Sabine Schnadt, Falk Schwendicke. Project conceptualization, administration, and funding acquisition: Birgit Ahrens, Thomas Holzhauser, Sabine Schnadt, Andreas Fickenscher, Falko Böhringer, and Kirsten Beyer. Project coordination by Birgit Ahrens and Kirsten Beyer. The latter are responsible for the study design, execution of the protocol, management, analysis, and interpretation of the data, writing reports, and submission to publication in close collaboration with all authors.

## FUNDING INFORMATION

SOLIDS‐By‐Kiss is part of the collaborative project Solids‐by‐Kiss. The project is supported by funds of the Federal Ministry of Food and Agriculture (BMEL) based on a decision of the Parliament of the Federal Republic of Germany via the Federal Office for Agriculture and Food (BLE) under the innovation support programme. Project code: 281A305A18, 281A305B18, 281A305C18, 281A305D18, 281A305E18, 281A305F18. The funding source had no role in the design of the trial; it will not have any role during the execution of the trial, on data management, analyses, interpretation, or publication of the results.

## CONFLICT OF INTEREST STATEMENT

B Ahrens receives research funding from the Federal Ministry of Food and Agriculture (BMEL); she reports participation in RCTs dealing with (hydrolysed) infant formulas for allergy prevention (HiPP); the latter outside the submitted work. K Beyer reports advisory board/consulting fees or speakers bureau from Aimmune Therapeutics, Bencard, Danone/Nutricia, DBV, Hycor, Infectopharm, Mabylon, Meda Pharma/Mylan, Nestle, Novartis, and ThermoFisher; and research grants from Aimmune, ALK, Danone/Nutricia, DBV Technologies, Hipp, Infectopharm, and Novartis outside the submitted work. K Blumchen reports advisory board/consulting fees or speaker's bureau from Aimmune Therapeutics, DBV Technologies, Novartis, Bencard Allergie, Stallergenes Greer, Thermofisher Scientific, Danone, Allergopharma, Mylan, Sanofi, Engelhard, ALK, Siemens Healthineers; and research grants from Novartis Pharma GmbH, Aimmune Therapeutics, DBV Technologies, Allergy therapeutics outside the submitted work.

## ETHICS STATEMENT

The trial was approved by the Ethics Committee of Charité – Universitätsmedizin Berlin (REC reference EA2/063/20) on 19 February 2021 and the Ethics Committee of the Goethe Universität Frankfurt a. M. (2021–482) on the 8th of November 2021 prior to the start. The presented protocol is based on protocol version 2.0 dated 04 October 2021. Amendments were added in the course of the trial.

## CONSENT

All caregivers give their full consent before their children are enrolled in the trial. The written informed consent must be given by both the mother and father, or the legal representatives. Please refer to Appendix S1 for information on: Protocol Amendments; Consent or assent; Additional consent provisions for collection and use of participant data and biological specimens; Confidentiality; Ancillary and post‐trial care; Dissemination plans; Plans to give access to the full protocol, participant‐level data, and statistical code.

## ROLES AND RESPONSIBILITIES

The trial is investigator‐initiated and sponsored by the Goethe University Frankfurt. The steering committee of the Solids‐by‐Kiss project (Birgit Ahrens, Kirsten Beyer, Thomas Holzhauser, Falko Böhringer, Andreas Fickenscher and Sabine Schnadt) will advise on the performance of the project throughout the duration of the trial. As this trial is an investigator‐initiated trial, only the principal investigators, sub‐investigators, and collaborators will be responsible and involved in the publications.

## DISCLAIMER

B Ahrens, L Schäfer, and T Holzhauser state: The views expressed in this work are their personal views and may not be understood or quoted as being made on behalf of or reflecting the position of the Paul‐Ehrlich‐Institut, the European Medicines Agency, or one of its committees or working parties.

## Supporting information


Appendix S1.
