# Supplementary material for: Pre‐chewing for weaning: A traditional method for allergy prevention? Rationale, study design, and methods of the open‐label trial—Solids‐by‐Kiss
Source: Pediatr Allergy Immunol. 2025 Aug 29;36(8):e70147. doi: 10.1111/pai.70147 (PMC12397717; doi:10.1111/pai.70147)
Supplement: Supplementary file 1 — Appendix S1. [file PAI-36-e70147-s001.docx]

**Title:**

**Pre-chewing for weaning – A traditional method for allergy prevention?** **Rationale, study design and methods of the open-label trial**

**Solids-by-Kiss**

**Supplementary data**

*Birgit Ahrens* ^1,5^*, Lara Meixner* ^2^*,* *Meral Sturmfels* ^1^*, Birgit Kalb* ^2^*, Anna Fischl* ^1^*, Falk Schwendicke* ^3^*, Katharina Blumchen* ^1^, Andreas Fickenscher ^4^, Laura Schäfer *^5^*, *Thomas Holzhauser ^5^*, Sabine Schnadt ^6^*, Kirsten Beyer* ^2,7^

*^1^ Goethe University Frankfurt, Dept. of Paediatrics, Division of Pneumology, Allergology, Infectious diseases and Gastroenterology, Frankfurt (Main), Germany*

*^2^ Department of Paediatric Respiratory Medicine, Immunology and Critical Care Medicine, Charité – Universitätsmedizin Berlin, corporate member of Freie Universität Berlin and Humboldt-Universität zu Berlin, Berlin, Germany*

*^3^ Conservative Dentistry and Periodontology, LMU Klinikum, LMU Munich, Munich, Germany*

*^4^ Fickenschers Backhaus GmbH, Münchberg, Germany*

*^5^ Paul-Ehrlich-Institut Allergology Division, Langen, Germany*

*^6^ Deutscher Allergie und Asthmabund e.V., Mönchengladbach, Germany*

*^7^ German Center for Child and Adolescent Health (DZKJ), partner site Charité Universität Berlin, Berlin, Germany*

**Participants Eligibility** **{10}**

The following inclusion criteria must be met: participants must be healthy, full-term born infants with a positive family history for atopic diseases (at least one parent or sibling suffers from atopic diseases such as atopic eczema, food allergy, allergic rhino conjunctivitis, allergic asthma). Caregivers will be excluded from the study if they do not have sufficient German language abilities due to the study information and study interactions being in German. For Group A and B, infants will be excluded from the interventional study if they have previously consumed hen’s egg, peanut, or hazelnut or suffer from a medically-diagnosed wheat allergy. Previous consumption of cow’s milk, e.g. as infant formula, is allowed, since infant formula is commonly produced using cow’s milk and formula-fed infants should not be excluded. Previous first contact with yoghurt or any other cow’s milk products is not an exclusion criterion either. The caregiver who will practice the pre-chewing of the ground biscuit will be excluded if they suffer from food allergies to hen’s egg, cow’s milk, peanut, wheat, or hazelnut. In addition, the parent/caregiver will be excluded in case of any acute, sub-chronic or chronic infectious disease (including caries) according to physician’s decision. Exclusion from study participation may also result from any other reported diseases or medical conditions in the caregiver’s medical history (for example, severe concurrent disease; infection; open wounds in the mouth; comorbidities such as acute trauma, serious oncological disease, serious renal disease, relevant cardiovascular disease, autoimmune diseases) or any other factors that may, in the opinion of the study physician, interfere with the ability to participate in the study, cause undue risk, or complicate the interpretation of data concerning child or caregiver.

**Table 1: Inclusion and Exclusion Criteria for Group A or Group B**

| **Inclusion criteria** | **Exclusion criteria** |
| --- | --- |
| For the infant | For the infant |
| - Healthy, full-term born infants - Positive family history for atopic diseases (at least one parent or sibling suffers from atopic diseases such as atopic eczema, food allergy, allergic rhino conjunctivitis, allergic asthma) | - Previously consumed hen’s egg, peanut, or hazelnut* - Suffering from a medically diagnosed wheat allergy |
| For the caregiver | For the caregiver |
| - No food allergies to hen’s egg, cow’s milk, peanut, wheat or hazelnut - No (acute, sub-chronic or chronic) infectious disease (including caries) according to physician’s decision - Mental and linguistic capability to follow the protocol requirements | - Any reported disease or medical condition (for example, but not limited to, severe concurrent disease, infection, open wounds in the mouth, comorbidity, e.g. acute trauma, serious oncological disease, serious renal disease, relevant cardiovascular disease, autoimmune diseases) or reasons that may, in the opinion of the study physician, interfere with the ability to participate in the study, cause undue risk, or complicate the interpretation of data concerning child or caregiver |

*Previous consumption of cow’s milk, e.g. as infant formula, is allowed, since infant formula is commonly produced using cow’s milk and formula-fed infants should not be excluded.

**Strategies to improve adherence to interventions {11c}**

During study performance the study teams will stay in regular contact with participants (in-person, email, or telephone) to monitor and encourage study adherence and to answer any questions as they arise. Phone calls will be scheduled two weeks after V1, V2, V3, and V4 (Group A) and two weeks after V1 as well as one interim call at week 12 (Group B). From V1 on, caregivers will constantly be encouraged to keep a weekly diary, recording any adverse events (AEs), concomitant medication, and diet. In addition, caregivers in Group A will be reminded to record if and how often they actually pre-chewed the ground biscuit before passing it to the child and to follow instructions and precautionary measures for administering the ground biscuit. Tolerability and palatability of solids introduction will be recorded likewise.

**Discontinuation or deviation from the intervention protocol {11b}; Plans to promote participant retention {*18b*}**

In case of discontinuation or deviation from the intervention protocol, all participants are encouraged to maintain the scheduled visits or phone calls. If for any reason a family is unable to complete the planned “biscuit increase” after 6 ± weeks (protocol deviation), a repetition of extension of the last level is offered. If the intervention is discontinued, participants are offered to attend the exit study visit, V5. Accordingly, the participants are then no longer in the PP group, but they remain in the full analysis set (FAS). Collection of all possible data is planned.

Outcomes {12}

## Presentation of all main secondary endpoints

- Compliance assessment/adherence of parents/caregivers conducting pre-chewing via questionnaire on performance and frequency: query on a weekly basis, if performed daily; 4-7 times per week; < 3 times/week (compliance defined as at least four days/week ≙ 57%)
- Composition of the oral microbiome and frequency of infant caries by one year of age (V5) according to the International Caries Detection and Assessment System (ICDAS, a clinical scoring system allowing detection and assessment of caries lesions and caries experience^30^)
- Frequency of IgE-mediated food allergies to at least one of the four foods (hen’s egg, cow’s milk, peanut, or hazelnut) after intervention by one year of age (V5) between Groups A and B
- Allergen-specific IgE and IgG4 to hen’s egg, cow’s milk, peanut, hazelnut, Ara h 2, Cor a 14, and wheat at V1 and V5 in comparison between groups
- Cumulative occurrence (frequency and severity) of immediate type allergic symptoms by year of age associated with/without the feeding procedure
- Number of IgE-mediated food allergies by one year of age
  - Prevalence of investigator diagnosed atopic dermatitis using modified Hanifin and Rajka criteria^31,32^ by one year of age
  - In case of existing AD: Change of SCORAD and change of EASIscore at V1 and V5^32-34^
- Diversity of the dermal microbiome (cheek, interscapular back, whole body) in infants with risk for atopic diseases in Group A vs. Group B after introduction of solids (V5)
- Prevalence of wheezing (defined as more than one separate episode of wheeze symptoms clinically observed either by the study investigator, or based on caregiver’s descriptions in the diary) at V1 and V5 in group comparison
- Cumulative Occurrence (frequency and severity) of respiratory and gastrointestinal infections (including oral infections) in comparison between groups
- Cumulative Occurrence (frequency and severity) of gastrointestinal symptoms other than infections (flatulence, stool colour and consistency, tolerability) in comparison between groups
- Occurrence of failure to thrive in comparison between groups

Participant timeline {13}

The duration will depend on the time of study entry. All participants of Group A will visit the study site at five timepoints (V1-V5). Participants of Group B will visit the study site at two timepoints (V1 and V5). The duration of the intervention period between V1 and V5 will be approximately 4-8 months, depending on the time interval between each next biscuit product. However, participants may start at V0 with or without V1 and V5 or only take part in the reference group at V5 (solo). This results in a maximum duration of approx. 12 months (depending on the individual age at enrolment: V0 = at birth, V1 = at about half a year old, V5 = at about one year old); singular visit by participation at V5(solo).

Depending on the necessity of an oral food challenge (V6) in sensitised infants at V5, the whole study duration may be extended for an additional 3- 6 months. Please see also Figure 1 and Table 3.

**Recruitment {15}**

This two-centre trial recruits participants in Germany at the Goethe University Frankfurt a. M., and at the Charité – Universitätsmedizin Berlin as well as within the child’s regular check-up appointments at primary care physicians in proximity of Frankfurt and Berlin, and via information leaflets. In addition, recruitment is supported by the patient organisation German Allergy and Asthma Association (DAAB, as collaboration partner), via its own newsletters and information circles.

## Continuation of Data collection methods {18}

*Skin swabs for microbiome analysis*

Skin swabs will be collected and preserved in DNA/RNA shield collection tubes containing preserving medium (DNA/RNA Shield; Zymo Research, Irvine, CA, USA). Swabs will be taken at the following sites of the infant: cheek (single sided), interscapular back, and whole body; mother: skin swabs will be taken at the Mamille/breast (single sided) only. Parents are advised to strictly avoid cleaning, washing or moisturising the skin for 24h previous to the skin swabs.

*Allergy diagnostic including Skin Prick Test (SPT) and blood sampling*

At V1 and V5 blood samples from the infant will be collected and stored for allergy diagnostics (including food-specific IgE and IgG4 to hen’s egg, cow’s milk, peanut, hazelnut, Ara h 2, Cor a 14, and wheat). Diagnostics will be measured using the ImmunoCAP Phadia™ 200 system (Phadia, Uppsala, Sweden). Blood samples will be stored for further analysis (e.g. of innate and adaptive immune responses). At V5 infants will be skin prick tested for hen’s egg, cow’s milk, hazelnut, peanut, dog, and house dust mite (Dermatophagoides pteronyssinus), with histamine and control solutions using commercial extracts (all extracts including positive and negative controls: ALK Abelló, Germany), and in accordance with standard clinical methods. If a food-specific sensitisation is detected at V5 (elevated food-specific IgE (> 0.1 kU/l) or positive SPT (wheel size > 3 mm)), a double-blind, placebo-controlled oral food challenge will be offered for evaluation of clinical relevance (optional visit V6). Consequently, parents will be advised to continue feeding the study product until food challenge performance.

*Double-Blind Placebo-Controlled Food Challenge (DBPCFC)*

Participants with suspected food allergy/sensitisation to the tested foods will be invited to undergo an oral food challenge, OFC (V6). The OFC will be performed as a DBPCFC in accordance with national guidelines^35^ using raw (pasteurised) hen’s egg, fresh cow’s milk, peanut, and hazelnut. This challenge involves a maximum of seven increasing dose steps administered at 30 min intervals using a semi-log scale (e.g. ranging from 3 mg to 3 g peanut protein followed by a cumulative dose of 4.5 g of peanut protein on another day if stepwise provocation was negative). Children with allergic reactions to raw hen’s egg or cow’s milk will be offered an additional OFC with heated hen’s egg/milk. An additional blood draw as well as skin swab, stool and saliva sample will be collected at this step. In case of a diagnosed food allergy, individualised dietary counselling will be carried out and further care in our outpatient clinics will be offered.

*Skin Barrier Permeability*

At the Berlin study site only, palms and palm lines of the infant will be visually assessed by photographic documentation at V1 and a measurement of the child’s transepidermal water loss (TEWL^36^ will be carried out at V1-V5. Measurements will be performed with the Tewameter TM Hex (Courage + Khazaka electronic GmbH, Germany). The water evaporation rate of the skin is determined indirectly by applying a sensor to the skin.

**Data management {19}**

Research Electronic Data Capture (REDCap) will be used as an electronic case report form (eCRF) to collect and manage the study data. REDCap is hosted at the Charité – Universitätsmedizin Berlin and provides an interface for data entry for clinicians. Data access and storage will follow the data security concept of the Charité, including firewalls at the campus level, institutional level, and individual computer level and password-protected access to all computers and folders that contain sensitive data.

**Methods: Monitoring**

**Composition of the data monitoring committee, its role, and reporting structure {21a}**

Trial monitoring to ensure compliance with good clinical practice will be conducted by the Trial Coordination Center (TCC), Department of Medicine 2, Hematology/Oncology, University Medicine, Goethe University Frankfurt to ensure the integrity of the trial. Data will be collected by the trained study personnel. The trial management committee will meet once a month and all collaboration partners, together with principal investigators and sub-investigators will meet twice a year to review trial conduct. In addition, the Ethics Committee of Charité - Universitätsmedizin Berlin and the Ethics Committee of University Medicine Frankfurt, Goethe University Frankfurt must approve any substantial amendments to the protocol or to the consent materials before implementation.

**Interim analyses {21b}**

No interim analyses for efficacy are planned.

**Auditing {23}**

An audit is not planned by the sponsor due to the nature of the study. The steering committee of the Solids-by-Kiss project will advise on the performance of the project throughout the duration of the trial. Trial monitoring to ensure compliance with good clinical practice will be conducted by the Trial Coordination Center (TCC), Department of Medicine 2, Hematology/Oncology, University Medicine, Goethe University Frankfurt to ensure the integrity of the trial.

**Protocol Amendments {25}**

The informed consent form must be revised whenever important new safety information is available, whenever the protocol is amended leading to changes in study procedures relevant for the patient, and/or whenever any new information becomes available that may affect participation in the trial.

**Consent or assent {26a}**

The consent procedure corresponds to the German national requirements. Both the conversations and the information brochure as well as the consent documents will be exclusively in German. The legal representative(s) of all participants must read, sign, and date the informed consent form before entering the study or undergoing any study-specific procedures. Before consent is given, the investigator or his/her representative will explain verbally the aim, method, source of funding, and the anticipated benefits and potential risks of the study to the parents; answer all questions regarding the study; and document the informed consent process.

**Additional consent provisions for collection and use of participant data and biological specimens {26b}**

As a point of opt-in consent included in the main study consent form, biological specimens are collected and partially stored for future analysis.

**Confidentiality {27}**

Participant’s privacy and confidentiality will be respected throughout the study. To ensure the protection of personal data, the national legal requirements including the EU General Data Protection Regulation (GDPR) regarding data confidentiality will be followed. Appropriate consent for collection, use, disclosure, and/or transfer (if applicable) of personal information must be obtained in accordance with local data protection laws. A unique participant identifier will be allocated to each participant and assigned chronologically prior to proceeding with study screening. These participant identifiers rather than names will be used to collect, store, and report participant information, including documentation in the eCRF. If the participant’s name appears on any other document (e.g. laboratory report), it must be removed from any copies of the document to be used elsewhere, e.g., uploaded to the eCRF. The investigator must retain records and documents, including signed informed consent forms, pertaining to the conduct of this study for 15 years after study completion and final publication. No records may be destroyed during the retention period without the written approval of the sponsor.

**Ancillary and post-trial care {30}**

Participants who developed a clinically-relevant food allergy after completion of the trial will receive individualised dietary counselling. Moreover, parents are given the option to contact either the outpatient clinic of the Department of Paediatrics, Division of Pneumology, Allergology, Infectious diseases and Gastroenterology, Goethe University Frankfurt or the Department of Paediatric Pulmonology, Immunology, and Critical Care Medicine, Charité – Universitätsmedizin Berlin for further routine consultation.

Test person and travel insurance is taken out for the duration of the trial

**Dissemination plans {31a}**

Besides the study protocol, publications are planned for the results in peer-reviewed journals. Results will also be communicated in lay language to participants and health care providers.

**Plans to give access to the full protocol, participant-level data, and statistical code {31c}**

Data will be available upon reasonable request with restrictions regarding scientific purpose and data protection. For further information please see the German Clinical Trials Register https://drks.de/search/en (DRKS00027255). Please see Additional file 1 for the SPIRIT Checklist.^24^
